# Tissue specific signature of HHV-6 infection in ME/CFS

**DOI:** 10.3389/fmolb.2022.1044964

**Published:** 2022-12-14

**Authors:** Francesca Kasimir, Danny Toomey, Zheng Liu, Agnes C. Kaiping, Maria Eugenia Ariza, Bhupesh K. Prusty

**Affiliations:** ^1^ Institute for Virology and Immunobiology, Julius-Maximilians-University of Würzburg, Würzburg, Germany; ^2^ HHV-6 Foundation, Santa Barbara, CA, United States; ^3^ Department of Cancer Biology and Genetics (CBG), Institute for Behavioral Medicine Research (IBMR), The Ohio State University, Columbus, OH, United States

**Keywords:** HHV-6, ME/CFS, EBV, epstein-barr virus, herpesvirus, viral pathology

## Abstract

First exposure to various human herpesviruses (HHVs) including HHV-6, HCMV and EBV does not cause a life-threatening disease. In fact, most individuals are frequently unaware of their first exposure to such pathogens. These herpesviruses acquire lifelong latency in the human body where they show minimal genomic activity required for their survival. We hypothesized that it is not the latency itself but a timely, regionally restricted viral reactivation in a sub-set of host cells that plays a key role in disease development. HHV-6 (HHV-6A and HHV-6B) and HHV-7 are unique HHVs that acquire latency by integration of the viral genome into sub-telomeric region of human chromosomes. HHV-6 reactivation has been linked to Alzheimer’s Disease, Chronic Fatigue Syndrome, and many other diseases. However, lack of viral activity in commonly tested biological materials including blood or serum strongly suggests tissue specific localization of active HHV-6 genome. Here in this paper, we attempted to analyze active HHV-6 transcripts in postmortem tissue biopsies from a small cohort of ME/CFS patients and matched controls by fluorescence *in situ* hybridization using a probe against HHV-6 microRNA (miRNA), miR-aU14. Our results show abundant viral miRNA in various regions of the human brain and associated neuronal tissues including the spinal cord that is only detected in ME/CFS patients and not in controls. Our findings provide evidence of tissue-specific active HHV-6 and EBV infection in ME/CFS, which along with recent work demonstrating a possible relationship between herpesvirus infection and ME/CFS, provide grounds for renewed discussion on the role of herpesviruses in ME/CFS.

## Introduction

Human herpesvirus 6 (HHV-6) including both HHV-6A and HHV-6B are ubiquitous herpesviruses that remain latent in most of the population. In a neurological context, HHV-6 is commonly associated with post-hematopoietic stem cell transplant encephalitis (Schmidt-Hieber et al., 2011), but it has additionally been identified as a potential etiological pathogen in several chronic neurological disorders (Komaroff et al., 2020). It is difficult to establish a causal relationship between HHV-6 and the implicated neurological disorders as the virus lies latent in a high proportion of infected individuals (Lusso et al., 1991). The question of how HHV-6 can be an etiological pathogen in diseases that not all who are infected with HHV-6 experience is one that has been meticulously navigated by researchers investigating chronic neurological disorders of elusive etiology, particularly those investigating myalgic encephalomyelitis/chronic fatigue syndrome (ME/CFS). Recent pandemic has sparked interest in HHV-6A reactivation as a putative cause of ME/CFS-like symptoms observed in Long-COVID.

ME/CFS is a complex, multi-system disorder characterized by neurological, metabolic, and immune dysfunctions (Syndrome, 2015). Neurological disturbances that have been linked to ME/CFS include loss of concentration and memory, headache, unrefreshing sleep, and unexplained muscle and joint pain (Bjørklund et al., 2020). Hypoactivation of hypothalamic-pituitary-adrenal (HPA) axis (Morris et al., 2017; Vangeel et al., 2018) and the basal ganglia (Miller et al., 2014) have been associated with ME/CFS, along with autoantibodies against serotonin (Ortega-Hernandez et al., 2009) and a reduction in serotonin transporters in the hippocampus (Cleare et al., 2005) and anterior cingulate (Yamamoto et al., 2004). A recent meta-analysis found that abnormalities in the brain stem are often reported in ME/CFS neuroimaging studies (Shan et al., 2020). Muscle pain is common in ME/CFS (Wyller, 2019), though the cause of this pain is not well understood and there are a variety of proposed explanations (Gerwyn and Maes, 2017). One possible explanation models pain in ME/CFS as a result of microglial accumulation in the L4-L6 dorsal horn of the spine (Yasui et al., 2014), but this model has yet to be tested in humans. ME/CFS brains have been found to have reduced white matter volume and abnormal fractional anisotropy in the right arcuate fasciculus (Zeineh et al., 2015).

HHV-6 and Epstein-Barr virus (EBV) have been hypothesized as etiological pathogens in ME/CFS (Ariza, 2021; Cox et al., 2022). However misconceptions of earlier studies justifying detection of viremia as a requirement to demonstrate a causal relationship between these viruses and ME/CFS has led to the doubts regarding involvement of herpesviruses in ME/CFS (Soto and Straus, 2000). Recent findings have demonstrated that expression of HHV-6 miRNAs during virus reactivation, in the absence of viral DNA replication, is enough to cause decreased mitochondrial functioning (Schreiner et al., 2020; Hennig et al., 2022) potentially leading to alterations in mitochondrial metabolism, host innate immune response. We and others have reported that HHV-6 viral DNA and RNA are detected at a very low copy number in blood and serum of ME/CFS patients suggesting the possibility of localized virus reactivation. To understand the potential tissue specific viral reactivation in ME/CFS patients, we carried out fluorescence *in situ* hybridization (FISH) analysis in postmortem tissue biopsies of a small cohort of ME/CFS patients (n = 3) and controls (n = 24) and found abundant transcription of HHV-6 miR-U14 in frontal lobe, basal ganglia, and spinal cord of ME/CFS patents. Our results confirm tissue-specific HHV-6 reactivation in ME/CFS patients and provide a ground for reconsidering a causal role of HHV-6 in various clinical anomalies observed in ME/CFS.

## Materials and methods

### Sample collection

Deidentified formalin-fixed and paraffin-embedded (FFPE) postmortem tissue biopsies were acquired from Cambridge Brain Bank, Cambridge University Hospitals, United Kingdom under NHS Research Ethics Committee Approval number 10/H0308/56. Ethics Commission of University of Würzburg did not require the study to be reviewed or approved by an ethics committee because all the tissue samples were obtained under a written agreement that allows the use of samples for a specific project pre-approved by the Brain biobank. Donors gave informed written consent for the use of brain tissue for research. Biopsy samples included tissues from 3 ME/CFS patients and 3 patients with other clinical diagnosis (anorexia, non-Hodgkin’s lymphoma, and breast cancer) as controls. Patients’ age, sex, clinical diagnosis, and tissue sample details are listed in Supplementary Table S1. Additionally, control biopsies from 21 more cases without having ME/CFS were acquired from Cambridge Brain Bank and were also included in this study (Supplementary Table S1).

### Fluorescence *in situ* hybridization (FISH)

FISH to detect HHV-6 miR-aU14 was carried out as described before (Descamps et al., 2021; Mysore et al., 2021).

### Dual staining for HHV-6 miR-aU14 and viral proteins

Followed by FISH, slides were permeabilized with 0.2% Triton-X100 in PBS for 30 min at room temperature. After 3 washes with PBS, slides were blocked with 10% FCS for 30 min at room temperature. Slides were incubated with primary antibodies within a humidity chamber for 1 h at room temperature, followed by 3 PBS washes. Subsequently, slides were incubated with secondary antibodies within a humidity chamber for 1 h at room temperature, followed by PBS wash. Dehydration of the slides was performed with ethanol. Slides were then mounted using a mounting medium. All antibodies were diluted in 2% FCS (Sigma-Aldrich, United States). Antibodies used for immunofluorescence studies include anti-HHV-6B U94, anti-HHV-6B OHV3, anti-HHV-6 gB, anti-HHV-6 p41 and anti-EBV dUTPase. EBV dUTPase antibody is a rabbit polyclonal antibody against the full-length protein. Purkinje cells were stained using anti-GFAP (astrocyte specific staining, AB5804, Millipore) and anti-Iba1 (microglia specific staining, Ab178846, Abcam) monoclonal antibodies. Imaging for viral miR-aU14 and viral proteins were carried out using a LSM 780 (Carl Zeiss AG, Germany) confocal fluorescence microscope using ZEN software.

## Results

### Active HHV-6 infection in brain biopsies of ME/CFS patients

HHV-6 displays neurotropism and has long been proposed to be associated with ME/CFS. Reactivation of the virus in the neuronal system could be a cause for clinical symptoms related to the disease. However, no studies have been carried out so far to investigate potential tissue-specific HHV-6 reactivation in ME/CFS patients. We analyzed FFPE-post-mortem tissue biopsies from 3 ME/CFS patients and three patients with other clinical diagnosis (anorexia, non-Hodgkin’s lymphoma, and breast cancer) for potential signature of HHV-6 infection by immunofluorescence analysis using antibodies against HHV-6B U94 (potential marker for viral latency), HHV-6B late protein OHV-3 (marker for active infection) and HHV-6 glycoprotein gB and p41 (markers for active infection).

**FIGURE 1:**
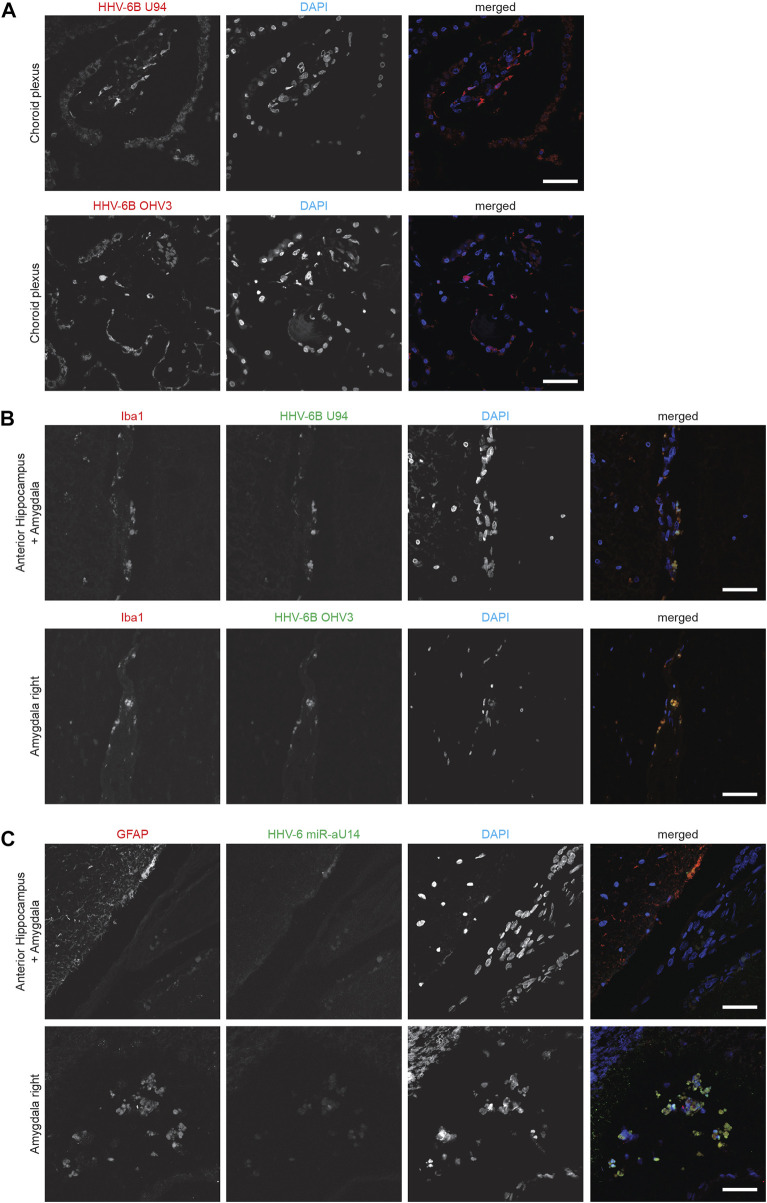
Representative fluorescence microscopy images of different human brain tissue types of ME/CFS patients. Tissues were co-stained with antibodies against HHV-6 specific proteins and/or neuronal tissue-specific marker and counter-stained for DAPI. **(A)** Human Choroid plexus tissues co-stained for HHV-6B U94 or OHV3, respectively. For each Panel images from the left to the right show markers for HHV-6 infection (red) and cell nuclei stained by DAPI (blue) and an overlay of the images. **(B)** Human anterior Hippocampus and Amygdala tissues co-stained for Iba1, HHV-6B U94 or OHV3. For each Panel images from the left to the right show astrocytes stained with Glial fibrillary acidic protein (GFAP) (red), markers for HHV-6 infection (green), cell nuclei stained by DAPI (blue) and an overlay of the images. **(C)** Human anterior Hippocampus and Amygdala tissues co-stained for Iba1 and HHV-6 miR-aU14. For each Panel images from the left to the right show astrocytes stained with GFAP (red), markers for HHV-6 infection (green), cell nuclei stained by DAPI (blue) and an overlay of the images. The scale bars represent 100 μm.

**FIGURE 2:**
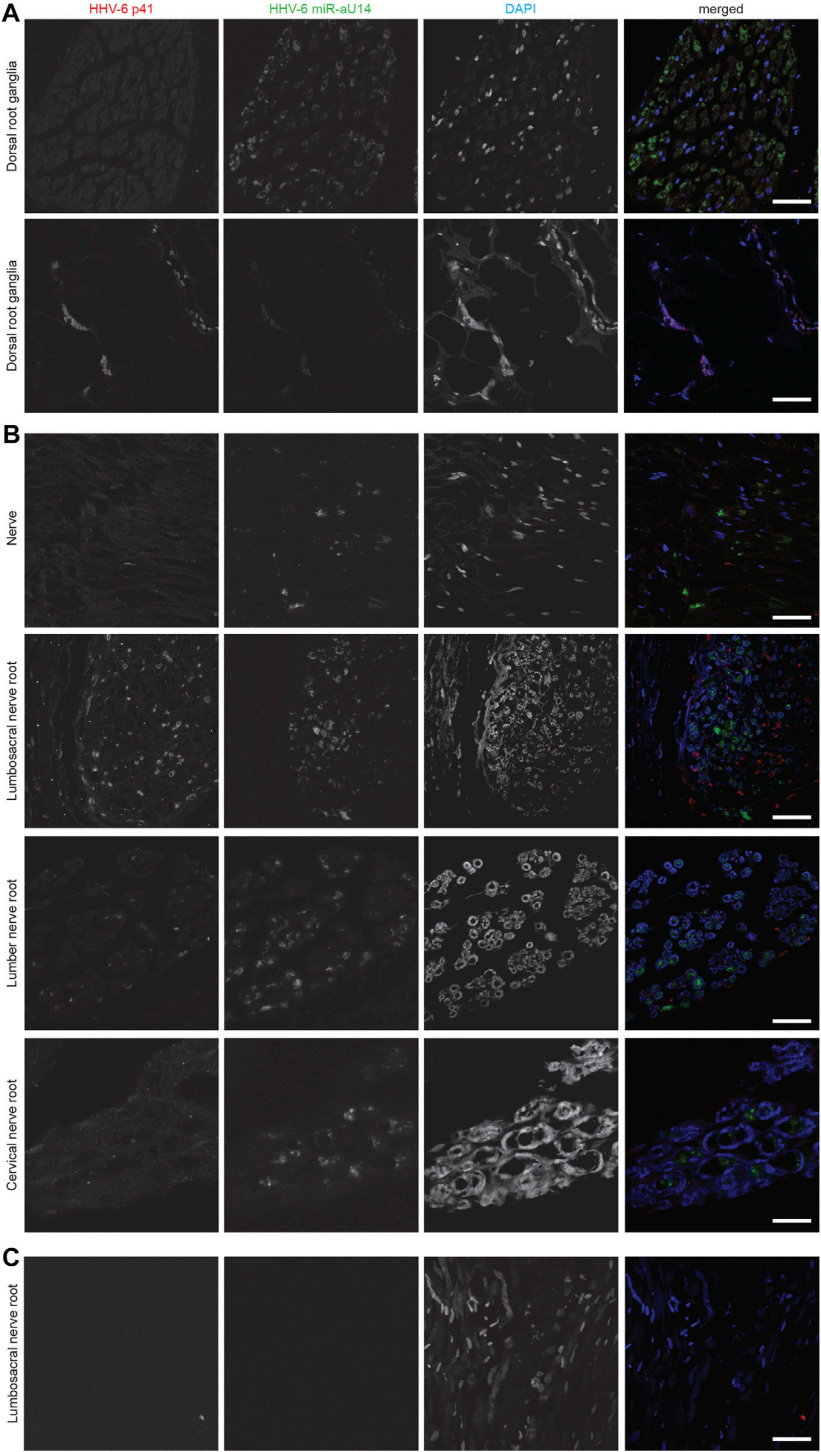
Representative fluorescence microscopy images of different human neuronal tissue types from two different ME/CFS patients and a control individual. For each panel images from the left to the right show HHV-6 p41 (red), a marker for active HHV-6A infection, miR-aU14 (green), cell nuclei stained with DAPI (blue) and an overlay of the images. **(A)** Human Dorsal root ganglia tissues of a ME/CFS patient. **(B)** Nerve, Lumbosacral nerve root, lumber nerve root and cervical nerve root tissues of a ME/CFS patient. **(C)** Lumbosacral nerve root tissue of a ME/CFS patient. The scale bars represent 100 μm.

HHV-6B U94 and OHV-3 antigens were found in Choroid plexus tissues from one of the ME/CFS patients (Figure 1A). Additionally, U94 protein was detected in Ionized calcium binding adaptor molecule 1 (Iba1) positive neuroglial cells within the anterior Hippocampus and left Amygdala of the patient but not in the right amygdala (Figure 1B). Additionally, FISH analysis detected viral miR-aU14 within GFAP-positive astrocytes within the same tissues (Figure 1C). Another patient biopsies showed positive staining for HHV-6 p41 antigen in cervical, lumbar, and sacral nerve roots (Figures 2A,B) (Supplementary Table S1). In contrast to these findings, the third ME/CFS patient was negative for all HHV-6 proteins (Figure 2C) (Supplementary Table S1) like the control group. Taken together HHV-6 reactivation was observed in two of 3 ME/CFS patients in selected tissue sections (Table 1).

**TABLE 1:** ME/CFS Patients with HHV-6 and EBV positive samples.

Patients and tissue types studied			Results of HHV-6 and EBV analysis					
Age	Sex	Sample	HHV-6 miR-aU14^a^	HHV-6B U94^b^	HHV-6B OHV-3^b^	HHV-6 gB^b^	HHV-6 p41^b^	EBV dUTPase^b^
24	Female	Choroid plexus	Negative	**Positive**	**Positive**	Negative	Negative	Negative
		Mid Brain	Negative	Negative	Negative	Negative	Negative	**Positive**
		Post. Hippocampus Left	Negative	Negative	Negative	Negative	Negative	**Positive**
		Post. Hippocampus Right	Negative	Negative	Negative	Negative	Negative	**Positive**
		Ant. Hippocampus + Amygdala Left	**Positive**	**Positive**	Negative	Negative	Negative	Negative
		Amygdala Right	**Positive**	Negative	**Positive**	**Positive**	Negative	Negative
		Dorsal Root Ganglia	**Positive**	Negative	Negative	Negative	Negative	Negative
		Dorsal Root Ganglia	**Positive**	Negative	Negative	Negative	Negative	Negative
43	Male	Temporal lobe/Hippocampus	Negative	Negative	Negative	Negative	Negative	**Positive**
		Cervical nerve root	**Positive**	Negative	Negative	Negative	**Positive**	Negative
		Lumbar nerve root	**Positive**	Negative	Negative	Negative	**Positive**	Negative
		Lumbar/Sacral nerve root	**Positive**	Negative	Negative	Negative	**Positive**	Negative
		Nerve	**Positive**	Negative	Negative	Negative	Negative	Negative
34	Female	Kidney	Negative	Negative	Negative	Negative	Negative	**Positive**
		Brain	Negative	Negative	Negative	Negative	Negative	**Positive**

^a^ HHV-6 miR-aU14 was detected using fluorescence *in situ* hybridization (FISH);

^b^ HHV-6 proteins (U94, OHV-3, gB and p41) and EBV dUTPase protein were detected by immunofluorescence analysis.

### Tissue specific localization of HHV-6 miRNA in ME/CFS

HHV-6 miRNA, miR-aU14 is expressed only during lytic HHV-6 infection and reactivation and is linked to disrupted mitochondrial function (Hennig et al., 2022). Because of their small size, miRNAs are often extremely stable and can serve as an ideal marker for virus reactivation studies (Descamps et al., 2021; Mysore et al., 2021). To study the presence of HHV-6 miR-aU14 in ME/CFS patient tissues, we carried out FISH analysis using FFPE tissue biopsies. HHV-6 p41 protein and miR-aU14 were observed within specific brain tissues of ME/CFS patients (Figure 2) (Table 1, Supplementary Table S1). Specifically, HHV-6 p41 was detected in the dorsal root ganglia of one of 3 ME/CFS patients (Figure 2A). Viral miR-aU14 was also found in the same patient in one of the two dorsal root ganglia samples analyzed (Figure 2A upper panel). Moreover, the same protein was detected in the Lumbosacral nerve root, lumbar nerve root, and cervical nerve root as well but not in the nerve tissue of another ME/CFS patient (Figure 2B). No viral miRNA was detected in the third ME/CFS patient. In summary, two out of the 3 ME/CFS patients showed the signature of ongoing HHV-6 infection/reactivation with viral miRNA production within localized neuronal cells of the brain.

### Absence of HHV-6 infection within cerebellum of ME/CFS patients

**FIGURE 3:**
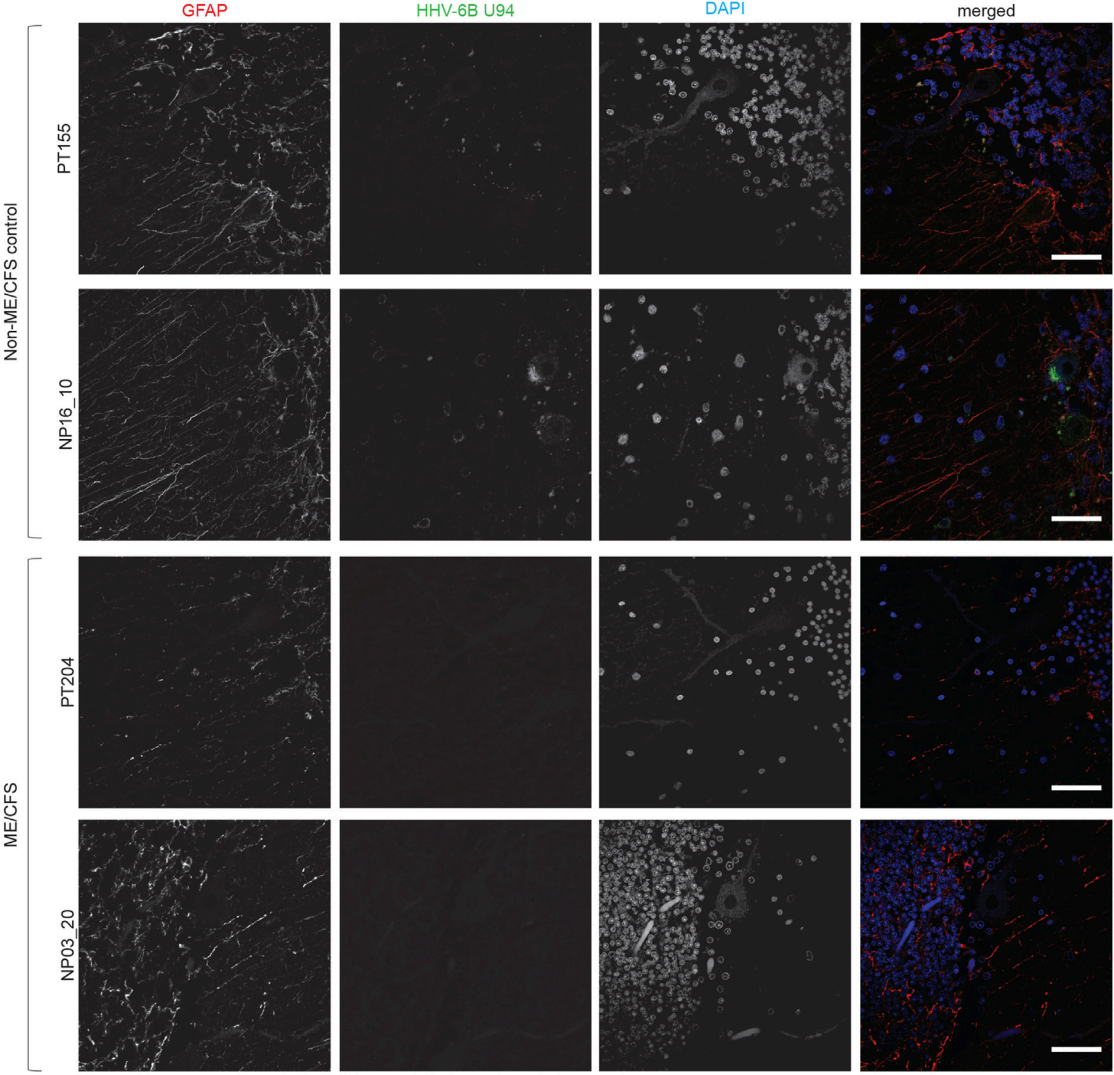
Absence of HHV-6 infection in cerebellum of ME/CFS patients. Representative images showing Immuno fluorescence analysis for HHV-6B U94 in cerebellum samples. Cerebellum samples were stained using antibody against HHV-6B U94 together with GFAP (marker for astrocytes). DAPI was used to counterstain DNA. Each panel represents a different ME/CFS patient or a non-ME/CFS control. The scale bars represent 100 μm.

We have previously shown association of active HHV-6 infection of cerebellum with mood disorders (Prusty et al., 2018). ME/CFS patients also suffer from psychological issues associated with depression. Therefore, we studied the cerebellum of these patients for potential HHV-6 infection. Interestingly, our study did not find any sign of HHV-6 infection within Purkinje neurons of ME/CFS patients (Figure 3). HHV6-U94 was not detected in cerebellum biopsies of all 3 ME/CFS patients. However, non-ME/CFS controls showed signs of HHV-6 U94 protein suggesting potential viral latency within this region (Figure 3). Analysis of HHV-6 miR-aU14, HHV-6B OHV-3, HHV-6 gB, and HHV6-p41 were all negative in the cerebellum biopsies of ME/CFS patients (Supplementary Table S1). These results suggest that HHV-6 infection within the cerebellum is not associated with any depressive physiology in ME/CFS patients.

### Co-infection of EBV in ME/CFS patients

**FIGURE 4:**
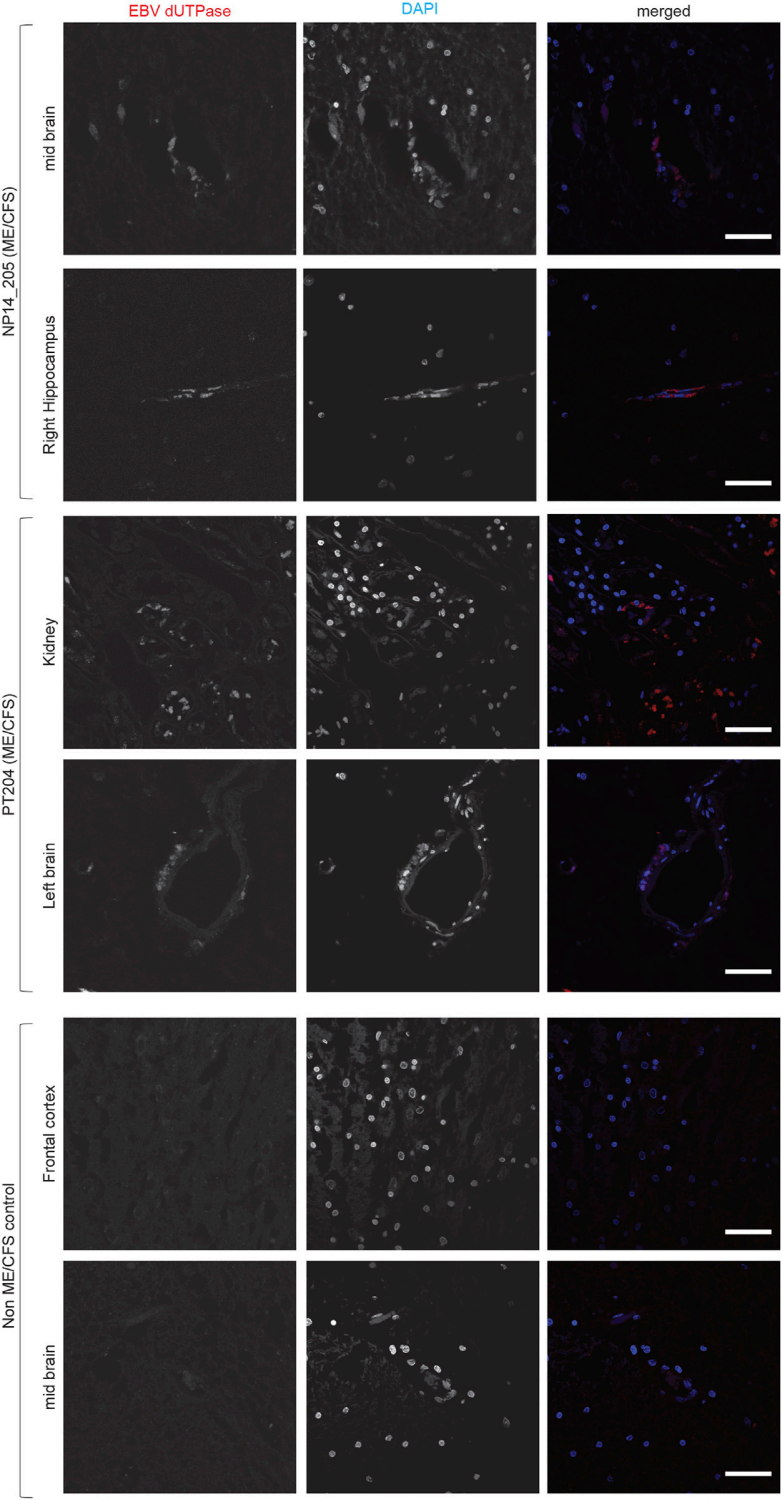
Co-infection of Epstein-Barr virus (EBV) in ME/CFS. Representative images showing Immuno fluorescence analysis for EBV dUTPase in multiple tissue samples from different ME/CFS patients and a non-ME/CFS control. Tissue samples were stained using a rabbit polyclonal antibody against EBV dUTPase. DAPI was used to counterstain DNA. The scale bars represent 100 μm.

Both HHV-6 and EBV infections are potential etiological pathogens for ME/CFS (Ariza, 2021; Cox et al., 2022). As active infection of HHV-6 was detected in multiple tissue biopsies from ME/CFS patients, we were interested in understanding potential co-EBV infection among these samples. EBV dUTPase has been detected in patients with chronic disorders. Hence, we analyzed post-mortem tissue biopsies for the presence of EBV dUTPase, which was abundantly detected in some tissue biopsies from ME/CFS patients, while all samples from non-ME/CFS control were negative for EBV dUTPase (Figure 4). Tissue samples from one of the ME/CFS patient showed positive staining for EBV dUTPase in the mid brain and right hippocampus (Figure 4) while the second patient showed positive EBV dUTPase staining only in hippocampus region. Interestingly, the third HHV-6 negative ME/CFS patient showed positive staining for EBV dUTPase in the left brain and kidney. None of the tissue biopsies were found to be dual positive for both HHV-6 and EBV. Overall, our results indicated that it is possible to have HHV-6 and EBV co-infection in ME/CFS patients.

## Discussion

HHV-6 has previously been detected in the meninges (Chapenko et al., 2016), frontal lobe (Lin et al., 2002; Hemling et al., 2003; Chapenko et al., 2016), olfactory tract (Chapenko et al., 2016), optic tract (Chapenko et al., 2016), hippocampus (Esposito et al., 2015; Huang et al., 2015; Chapenko et al., 2016), and white matter (Cermelli et al., 2003; Opsahl and Kennedy, 2005) of the brain. Symptomatic HHV-6 is associated with neuroinflammation when identified in the brain (Komaroff et al., 2020). In addition to ME/CFS, active HHV-6 in the brain has been linked to mesial temporal lobe epilepsy (MTLE), multiple sclerosis (MS), and Alzheimer’s disease (AD).

MTLE is the most common form of epilepsy (Engel Jr, 2001). Active HHV-6A/B infection is more common in the mesial temporal lobe in MTLE patients than patients with other forms of epilepsy (Fotheringham et al., 2007) and HHV-6B positivity in astrocytes of the hippocampus has been associated with MTLE (Niehusmann et al., 2010; Li et al., 2011; Esposito et al., 2015; Huang et al., 2015). A potential mechanism of HHV-6 involvement in the MTLE could be a vicious cycle of infection inducing neuroinflammation which in turn increases viral reactivation and further inflammation (Tamai et al., 2017; Kwon et al., 2018; Bartolini et al., 2019). This explanation is also useful in hypothesizing HHV-6’s role in MS, with the addition of several other findings. HHV-6 is found in higher levels in MS plaques compared to other areas of the brain (Friedman et al., 1999; Cermelli et al., 2003; Opsahl and Kennedy, 2005) and HHV-6 positivity in oligodendrocytes and microglia of the white matter has been associated with MS (Friedman et al., 1999; Blumberg et al., 2000; Goodman et al., 2003). The ability to induce neuroinflammation by different mechanisms makes HHV-6 a potential etiological pathogen for AD (Reynaud et al., 2014; Eimer et al., 2018; Bortolotti et al., 2019), though findings supporting this association have been disputed (Jeong and Liu, 2019; Allnutt et al., 2020). HHV-6 positivity in the frontal cortex has been associated with AD (Lin et al., 2002; Hemling et al., 2003) in some cases.

Dysregulation in the frontal lobe, basal ganglia, and dorsal root ganglia (DRG) may be responsible for several characteristic symptoms of ME/CFS. The cognitive slowing observed in ME/CFS has been linked to hypoactivation of the frontal lobe (Zinn et al., 2018), the fatigue observed in ME/CFS has been linked to hypoactivation of the basal ganglia (Miller et al., 2014), and the unexplained muscle pain observed in ME/CFS has been linked to inflammation or pressure on or around the DRG (Hulens et al., 2021). Active HHV-6 has been found in the frontal lobe (Hemling et al., 2003; Chapenko et al., 2016), basal ganglia (Achim et al., 1994), and dorsal root ganglia (Hüfner et al., 2007). Active HHV-6 in these tissues has been associated with inflammation in the frontal lobe (Chapenko et al., 2016) and basal ganglia (Achim et al., 1994; Crawford et al., 2007). While active HHV-6 has been identified in the DRG and was found to increase the susceptibility of the sensory ganglia to alpha-herpesvirus infection (Hüfner et al., 2007), a clear link between symptomatic HHV-6 infection and dysregulation in the dorsal root ganglia has yet to be made. In context with one another and the results of the present study, these findings indicate that active HHV-6 infection in the frontal lobe, basal ganglia, and dorsal root ganglia may lead to several characteristic symptoms of ME/CFS, and future studies should elucidate these potential links.

Like many other herpesviruses, HHV-6 and HHV-7 display neurotropism. HHV-6 infection and reactivation has been shown in astrocytes (Donati et al., 2005), glial cells (Cassiani-Ingoni et al., 2005) and Purkinje neurons providing strong evidence that link HHV-6 infection to various neurological disorders. In this study, using FISH, we have found miR-aU14 in the axons of the spinal cord in ME/CFS patients (Figure 2) but not in controls, suggesting HHV-6 might also undergo retrograde transport within the axons like HSV-1 (Bearer et al., 2000).

The present finding of active HHV-6 infection in the cervical nerve root may propose a potential pathophysiological mechanism for a series of findings by Matsui et al., In 2012, Matsui et al. proposed a novel syndrome called “cervical neuro-muscular syndrome”, which established a link between treating cervical muscle lesions and the alleviation of autonomic dysfunction in ME/CFS (Matsui et al., 2012), which was followed in 2020 with a study establishing a relationship between cervical muscle lesions and parasympathetic nervous system dysfunction (Matsui et al., 2020). These findings were elucidated in a 2021 study in which Matsui et al. observed a relationship between treatment of cervical muscle lesions with low frequency electrical stimulation, reduction in severity across a range of ME/CFS symptoms, and reduction of pupil diameter in patients whose symptoms improved (Matsui et al., 2021). Taken together, these findings indicate a relationship between cervical muscle lesions, severity of ME/CFS symptoms, and autonomic dysfunction in ME/CFS, which provide intriguing context for the present identification of active HHV-6 infection in the cervical nerve roots of ME/CFS patients.

Additionally, several recent studies have identified higher copy numbers of HHV-6 and EBV in ME/CFS patients compared to healthy controls (Fevang et al., 2021; Lee et al., 2021; Gravelsina et al., 2022). Lee et al. observed an association between HHV-6B and HHV-7 viral load and severity of ME/CFS symptoms by analyzing salivary viral load. Gravelsina et al. found a higher viral load of HHV-6B in peripheral blood mononuclear cells (PBMC) corresponding to the severity of ME/CFS symptoms, with 84.2% of patients with severe ME/CFS having a load greater than 1,000 copies per million PBMC compared to 57.1% of patients with mild ME/CFS and 11.1% of health controls (Gravelsina et al., 2022). Fevang et al. found a higher average copy number of EBV in the peripheral blood of ME/CFS patients compared to healthy controls, however the difference was not statistically significant (Fevang et al., 2021).

EBV has also been associated with several neurological disorders ranging from inflammatory conditions such as encephalitis and encephalomyelitis to neurodegenerative diseases such as Parkinson’s Disease and Alzheimer’s Disease (Zhang et al., 2021). Furthermore, a recent study suggested that EBV was the leading cause of MS, a chronic inflammatory demyelinating disease of the central nervous system (Bjornevik et al., 2022). The results of the present study support the results of previous studies suggesting that the EBV dUTPase may contribute to the neurological symptomology observed in some patients with ME/CFS (Williams Ph et al., 2019).

Unlike many other neurological diseases, ME/CFS is not associated with high rate of mortality. Because of this reason, it is difficult to obtain tissue biopsies from patients to carry out these types of studies. Small sample size is one of the key limitations of our study. Despite the small sample size, we aim to present the findings of active HHV-6 and EBV in the tissues of ME/CFS patients, and its absence in healthy controls, to further reason renewed discussion and interest in the role of herpesviruses in ME/CFS.

## Data Availability

The raw data supporting the conclusion of this article will be made available by the authors, without undue reservation.
